# Breast Phyllodes Tumor: A Tumor With Unpredictable Clinical Behavior

**DOI:** 10.7759/cureus.37537

**Published:** 2023-04-13

**Authors:** Ana Isabel Tomé, Joana Figueiredo, Sofia Carralas Antunes, Madalena Trindade, Daniel Travancinha

**Affiliations:** 1 Department of Obstetrics and Gynecology, Hospital Garcia de Orta, Almada, PRT; 2 Department of Pathology, Hospital Garcia de Orta, Almada, PRT; 3 Department of Surgery, Hospital Garcia de Orta, Almada, PRT

**Keywords:** adjuvant treatment, pathology, phyllodes tumor, fibroepithelial neoplasms, breast

## Abstract

Phyllodes tumors are uncommon biphasic breast tumors with a wide range of clinical behaviors. The distinction between a phyllodes tumor and a fibroadenoma can be difficult. The diagnosis of phyllodes tumor should be suspected in all women who present with a rapidly growing breast lump. Based on the histological characteristics, the World Health Organization (WHO) classifies phyllodes tumors as benign, borderline, or malignant. The risk of recurrence and metastatic potential varies based on histological features. Wide excision or mastectomy is the standard of care ensuring histologically clear margins. Despite the grading criteria defined by the WHO, the management of phyllodes tumors continues to be a challenge.

We report the case of a 48-year-old woman who presented to the emergency department with a large and ulcerated phyllodes tumor of the left breast. The tumor size did not allow conservative surgery. The final diagnosis of a borderline phyllodes tumor was made, and, in this case, the patient did not undergo adjuvant treatment.

## Introduction

Phyllodes tumors are rare fibroepithelial neoplasms of the breast characterized by a leaflike appearance with greater cellular stroma than fibroadenomas. They represent 2-3% of all fibroepithelial breast tumors and fewer than 1% of all breast tumors [1,2]. Preoperative diagnosis of phyllodes tumors based on clinical and pathological features is a challenge in part considering the limitations of core biopsy specimens [3]. According to the World Health Organization (WHO), phyllodes tumors are classified as benign, borderline, or malignant based on the presence of stromal cellularity and atypia, mitotic activity, stromal overgrowth, and tumor margins. Complete surgical excision is the standard of care for all histological grades; however, further decision-making regarding adjuvant treatment remains unclear [4,5].

## Case presentation

**Figure 1:**
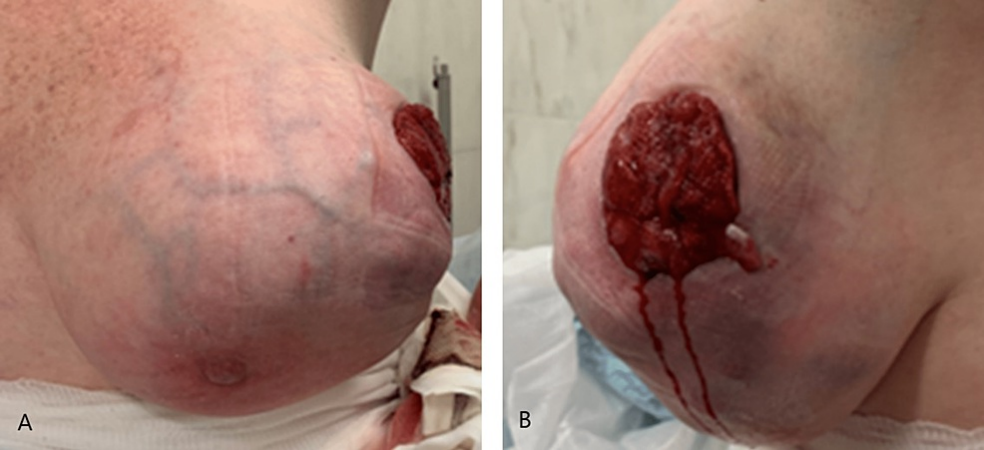
Large tumor of the left breast with ulceration of the upper outer quadrant (A: anterior view; B: lateral view).

**Figure 2:**
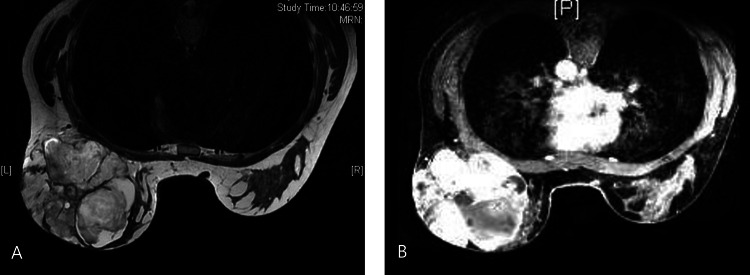
(A, B) Breast MRI showing a large tumor with irregular walls with T1-weighted image with high intensity and T2 image with low intensity. A rapid enhancement pattern is evident.

A 48-year-old woman, with an unremarkable medical history, presented to the emergency department during the COVID-19 pandemic due to an extensive and ulcerated breast lesion. She had been evaluated 18 months before in our tertiary hospital outpatient department for a left breast lump measuring 6.7 × 4.5 cm. A core needle biopsy was performed, and it showed a fibroadenoma with pericanalicular pattern with myxoid stromal alterations. The patient refused surgical treatment at the time. Physical examination revealed a lobulated mass that occupied almost the entire breast with superficial bulging veins and an ulcerated skin area at the outer quadrant (Figure 1). The patient underwent a core needle biopsy and complementary evaluation with axillary ultrasound, breast MRI (Figure 2), and CT scan of the chest/upper abdomen.

**Figure 3:**
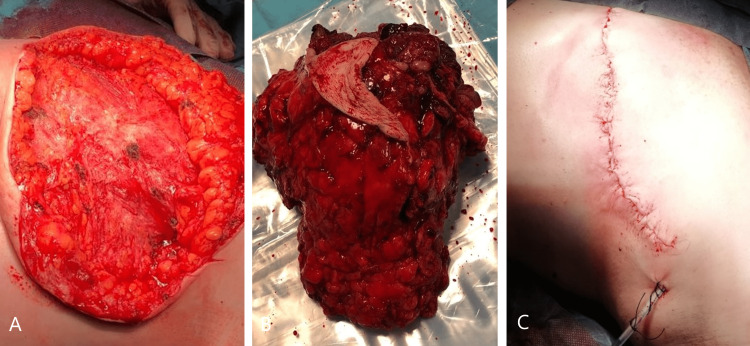
Left mastectomy: intraoperative images showing the large wall defect (A), the mastectomy specimen (B), and wound closure (C).

The pathology report was concordant with fibroepithelial neoplasia, not being able to exclude phyllodes tumor. Secondary lymph node or other organ involvement was not identified. A left mastectomy was performed, with no possibility of immediate reconstruction due to local conditions (Figure 3).

**Figure 4:**
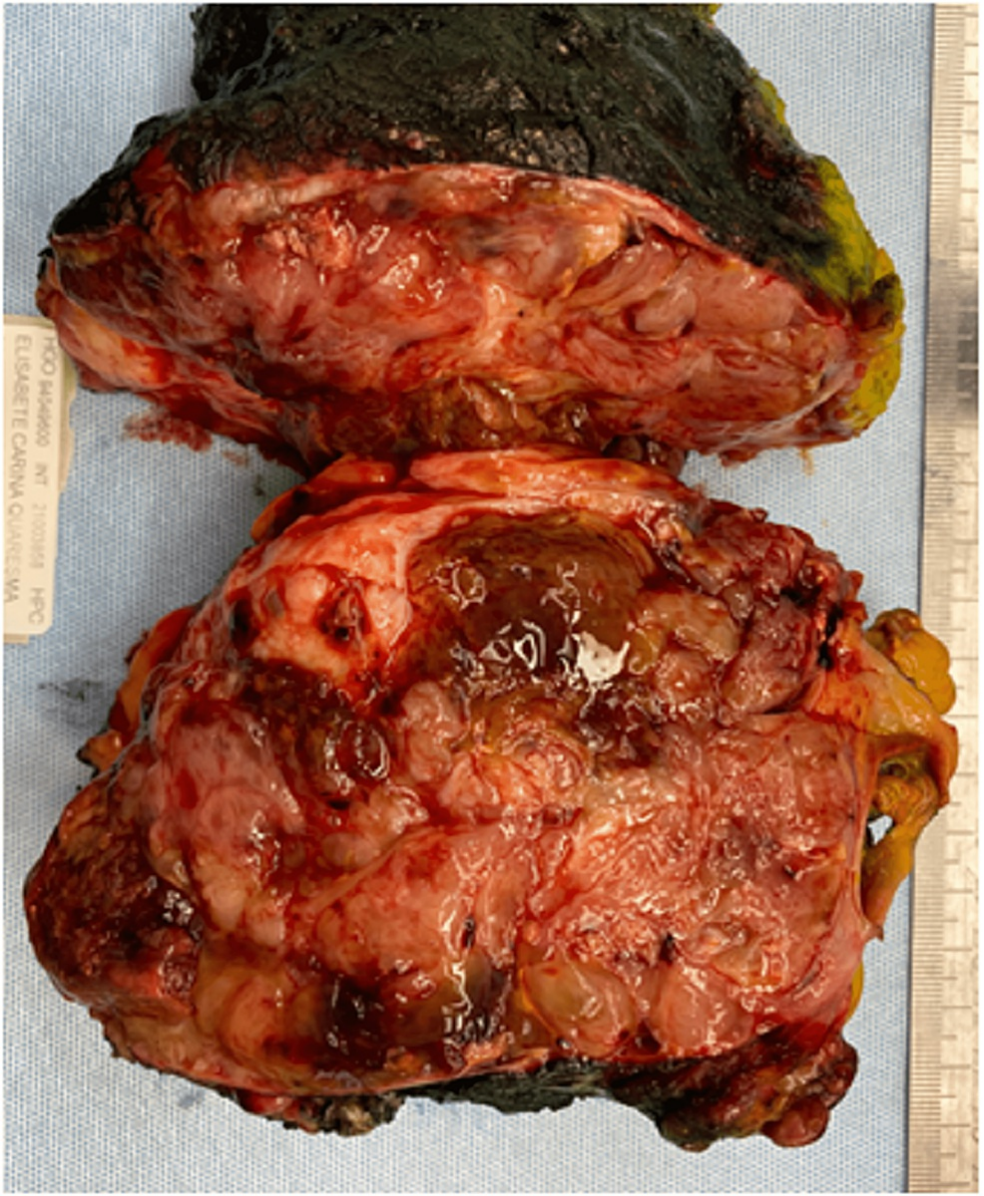
Mastectomy specimen (size: 20 × 13 × 6 cm; weight: 1.028 kg). The cut surface showing a well-circumscribed multinodular solid gray tumor with necrotic foci.

**Figure 5:**
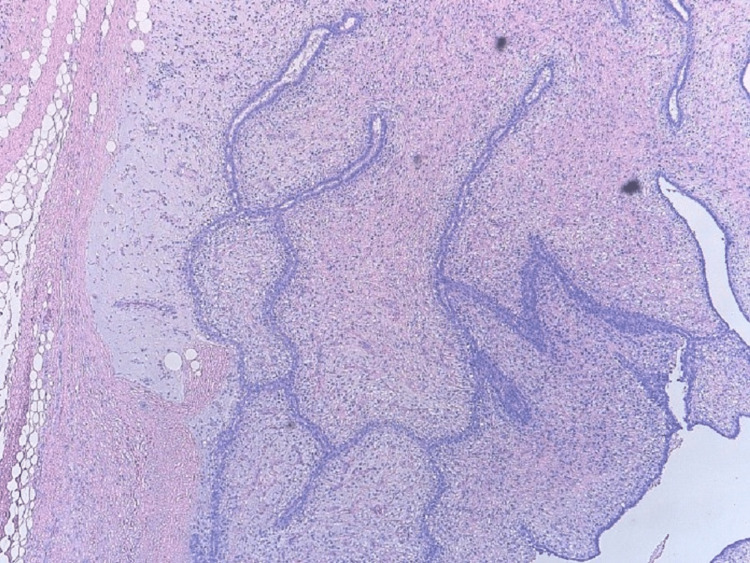
Histology with hematoxylin and eosin (4×) of the mastectomy specimen showing biphasic proliferation with epithelial and stromal components and a leaf-like pattern and clefts.

**Figure 6:**
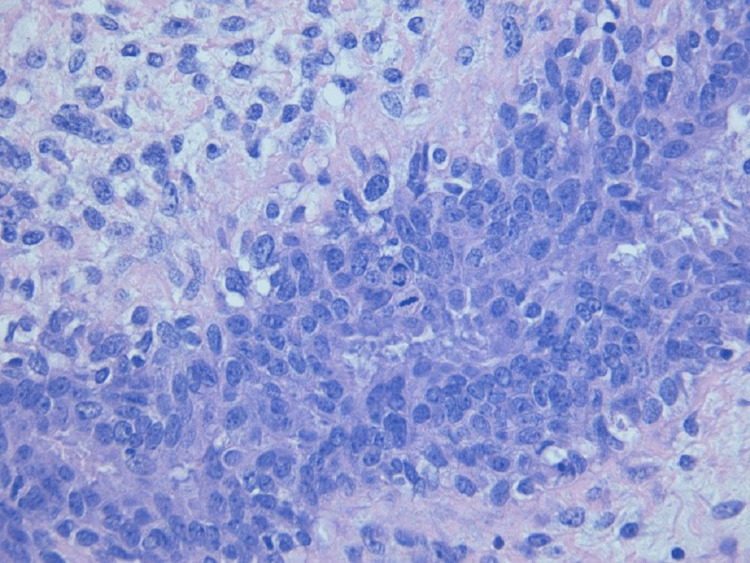
Histology with hematoxylin and eosin (4×) with a zoomed-in view of Figure 5 with evident mitosis (40×).

Grossly, the mastectomy specimen revealed a well-circumscribed multinodular solid tumor, with necrotic foci that measured 12 × 12 × 8 cm and had a deep free margin (Figure 4). Microscopically, it presented a biphasic proliferation with epithelial and stromal components, as well as a leaf-like pattern and clefts and a benign chondroid heterologous element. It had expansive growth, with mild-to-moderate nuclear atypia and 10 mitosis/10 high-power fields. Margin status revealed a free deep margin (1 mm), coincidental with internal, superior, and superficial margins (Figures 5, 6). These histological parameters defined the lesion as borderline [1]. Adjuvant treatment was not performed due to patient refusal. The patient remains disease free 22 months after surgery.

## Discussion

Breast phyllodes tumors are rare fibroepithelial tumors that account for fewer than 1% of primary breast cancers [1-3]. With a wide biological spectrum, these tumors range from benign lesions, with difficulty in making an initial differential diagnosis with fibroadenomas, to malignant lesions, with the capacity for recurrence and metastasis [6-8]. Our patient had a previous diagnosis of a pericanalicular fibroadenoma with stromal alteration that should have been further characterized but she refused surgical excision after initial evaluation. A multidisciplinary approach that includes integrated clinical, imaging, and pathological evaluation is essential for the correct diagnosis of phyllodes tumor and to determine the optimal therapeutic strategy [8-10]. According to the WHO classification [4], phyllodes tumors can be classified as benign, borderline, or malignant based on histologic features. In this case, the delay in seeking medical care was associated with poor aesthetic outcomes because the breast-to-tumor ratio did not allow a conservative approach and compromised margins width. The final diagnosis was a borderline phyllodes tumor with clear but narrow margins. Adjuvant radiotherapy was not administered in this case due to patient refusal. Nevertheless, the minimally acceptable margin after surgical resection of phyllodes tumor remains controversial even if mastectomy is performed, and the need for adjuvant radiotherapy should be determined on an individual basis [11-13]. Some studies have also shown that adjuvant radiotherapy does not improve survival in comparison to surgery alone [6,11]. Considering the lack of evidence-based treatment recommendations and the unpredictable clinical behavior of phyllodes tumors, close follow-up of patients is crucial to achieving the best outcomes.

## Conclusions

The differential diagnosis between fibroadenoma and phyllodes tumor is challenging and can be difficult on core biopsy. Because the mainstay of treatment of phyllodes tumors is complete surgical excision, early diagnosis allows more conservative surgery improving aesthetic outcomes and patient satisfaction. As there are no prospective randomized data supporting the use of adjuvant therapy for phyllodes tumors, it should be considered on a case-by-case basis.
